# Neutralization of TSLP Inhibits Airway Remodeling in a Murine Model of Allergic Asthma Induced by Chronic Exposure to House Dust Mite

**DOI:** 10.1371/journal.pone.0051268

**Published:** 2013-01-02

**Authors:** Zhuang-Gui Chen, Tian-Tuo Zhang, Hong-Tao Li, Fen-Hua Chen, Xiao-Ling Zou, Jing-Zhi Ji, Hong Chen

**Affiliations:** 1 Department of Pulmonary Diseases, The Third Affiliated Hospital of Sun Yat-Sen University, Institute of Respiratory Diseases of Sun Yat-Sen University, Guangzhou, China; 2 Department of Pediatrics, The Third Affiliated Hospital of Sun Yat-Sen University, Guangzhou, China; French National Centre for Scientific Research, France

## Abstract

Chronic allergic asthma is characterized by Th2-typed inflammation, and contributes to airway remodeling and the deterioration of lung function. However, the initiating factor that links airway inflammation to remodeling is unknown. Thymic stromal lymphopoietin (TSLP), an epithelium-derived cytokine, can strongly activate lung dendritic cells (DCs) through the TSLP-TSLPR and OX40L-OX40 signaling pathways to promote Th2 differentiation. To determine whether TSLP is the underlying trigger of airway remodeling in chronic allergen-induced asthma, we induced allergic airway inflammation in mice by intranasal administration of house dust mite (HDM) extracts for up to 5 consecutive weeks. We showed that repeated respiratory exposure to HDM caused significant airway eosinophilic inflammation, peribronchial collagen deposition, goblet cell hyperplasia, and airway hyperreactivity (AHR) to methacholine. These effects were accompanied with a salient Th2 response that was characterized by the upregulation of Th2-typed cytokines, such as IL-4 and IL-13, as well as the transcription factor GATA-3. Moreover, the levels of TSLP and transforming growth factor beta 1 (TGF-β1) were also increased in the airway. We further demonstrated, using the chronic HDM-induced asthma model, that the inhibition of Th2 responses via neutralization of TSLP with an anti-TSLP mAb reversed airway inflammation, prevented structural alterations, and decreased AHR to methacholine and TGF-β1 level. These results suggest that TSLP plays a pivotal role in the initiation and persistence of airway inflammation and remodeling in the context of chronic allergic asthma.

## Introduction

Allergic asthma is a common respiratory disease caused by chronic exposure to environmental aeroantigens like house dust mite (HDM), with the hallmark of airway chronic inflammation and structural alterations [1]–[3]. This chronic inflammation driven by Th2 responses is considered to be the underlying cause of damage to the airway epithelium. This damage is characterized by the elevated expression of TGF-β1 and ultimately results in subepithelial fibrosis, goblet cell hyperplasia, smooth muscle incrassation, and peribronchial collagen deposition, collectively referred to as airway remodeling [4], [5]. Airway remodeling is associated with a dysregulated repair process, and contributes to the physiological subphenotypes of irreversible or partially reversible airflow obstruction and progressive decline in lung function [6].

Several groups have demonstrated that airway remodeling is likely driven by Th2 responses [7]–[10]. The development of airway remodeling, including goblet cell hyperplasia and subepithelial fibrosis, was demonstrated to be dependent on Th2 responses [8]. Mice that are deficient in the genes that encode Th2 cytokines IL-4 and IL-13 were completely protected from developing airway remodeling and sustained airway hyperreactivity (AHR) following chronic allergen exposure [9]. Furthermore, Th1/Th2 homeostasis was conditioned by T-bet and GATA-3, the key transcription factors for naïve T cell differentiation toward Th1 and Th2 cell, respectively [10]–[12]. A shift in Th1/Th2 homeostasis to the Th2 responses caused airway wall structural remodeling. For example, in transgenic mice that overexpress GATA-3, the Th1/Th2 balance was shifted to Th2, with the result that structural alterations appeared in airway tissue. In contrast, in mice that overexpress T-bet, the Th1/Th2 balance was shifted to Th1, and structural remodeling of airway walls was prevented following allergen exposure [10]. However, the initiating factor that links airway inflammation to remodeling in chronic asthma remains unclear.

The airway epithelium is a pivotal regulator of innate and Th2 immunity, which has a central role in asthma pathogenesis [13], [14]. As an epithelium-derived cytokine, thymic stromal lymphopoietin (TSLP) represents a master switch at the interface between environmental allergens and pulmonary allergic immunologic responses [15]. TSLP was demonstrated to be a necessary and sufficient factor for the initiation of allergic airway inflammation by contacting lung dendritic cells (DCs) [16]. The OX40 ligand (OX40L) was found to be the TSLP-induced surface marker on DCs that mediated inflammatory Th2 cell differentiation [17]. TSLP-activated DCs upregulated OX40L expression, which then interacted with OX40 on T cells, resulted in the polarization of naïve T cells toward the Th2 pathway. This sequence of events resulted in the production of Th2 cytokines, such as IL-4 and IL-13, as well as TNF-α [18], [19]. In mice, TSLP overexpression led to spontaneous airway inflammation and an asthma phenotype [20], whereas mice lacking the TSLP receptor (TSLPR) exhibited substantially blunted allergic airway inflammation [21]. The local application of anti-TSLPR Ab prevented Th2-mediated airway inflammation [22]. Thus, TSLP appears to be a critical and essential factor in the context of allergic asthma. However, whether the T-bet/GATA-3 bias in asthmatic mice may be altered by blocking TSLP, thereby inhibiting airway remodeling, is unknown.

In the present study, the role of TSLP in the pathogenesis of airway remodeling in chronic allergic asthma was investigated using a chronic HDM-induced mouse model. The association between TSLP and the presence of on-going sustained airway inflammation and remodeling was also examined. We observed that TSLP is a pivotal contributor to airway remodeling in mice with chronic allergen-induced asthma. The local blockage of TSLP inhibited Th2-typed responses, including modulating the bias of T-bet/GATA-3 and reducing the levels of Th2-associated cytokines, such as IL-4 and IL-13. This inhibition of TSLP therefore eliminated established airway inflammation and prevented airway structural remodeling.

## Materials and Methods

### Animals

**Figure 1:**
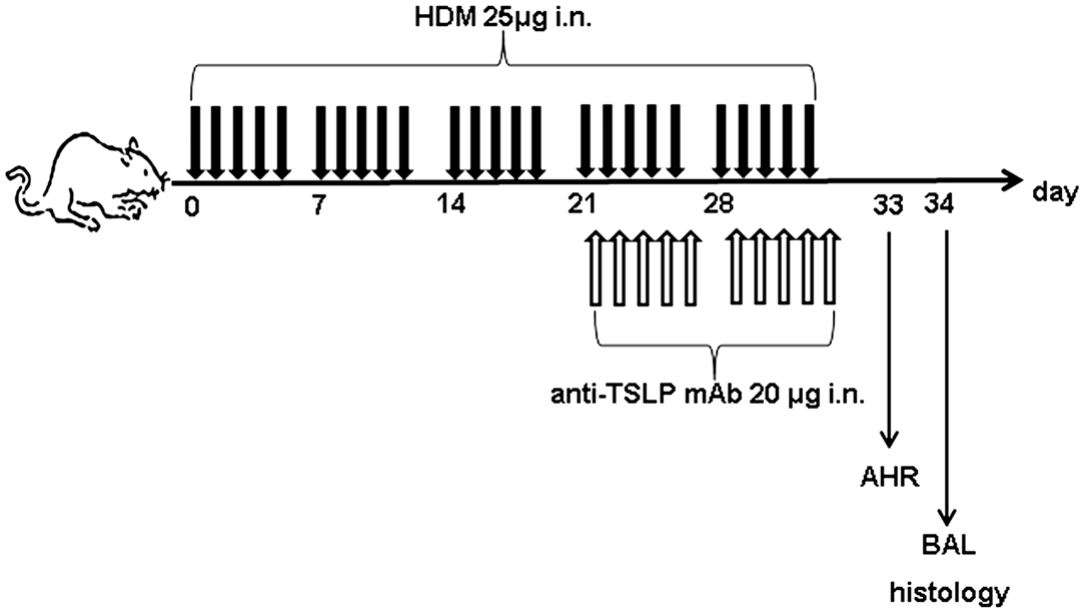
Experimental protocols for chronic HDM-induced asthma in mice. BALB/c mice received intranasal HDM on 5 consecutive days per week for up to 5 consecutive weeks. The negative control mice were intranasally administered saline daily on the same schedule. For the TSLP blocking experiment, the mice were intranasally administered anti-TSLP mAb prior to receiving HDM from the 4^th^ week to the 5^th^ week. The isotype control mice were intranasally administered vehicle IgG prior to receiving HDM on the same schedule. The mice were assessed by measuring the AHR to methacholine and baseline lung function on day 33 and then sacrificed with an over dose anaesthetized, BAL was performed, and the lungs were dissected for histological analysis 24 hours after the AHR measurement.

Female BALB/c mice (6–8 weeks old) were purchased from Shanghai Slac Laboratory Animal Centre and were housed under specific pathogen-free conditions. The animals were maintained in a 12-hour light-dark cycle and were provided food and water ad libitum. The positive control mice were exposed to purified HDM whole-body extracts (Greer Laboratories, Lenoir, North Carolina, USA) intranasally (25 μg HDM protein in 10 μl of saline) under inhaled anesthesia (isoflurane; Baxter, Kista, Sweden) for five consecutive days, followed by two days rest for five consecutive weeks [23]. The negative control mice were intranasally administered 10 μl of saline daily on the same schedule. No exogenous adjuvant was given at any time. For the TSLP blocking experiment, the anaesthetized mice received an intranasal (i.n.) administration of 20 μg of anti-TSLP mAb (MAB555; R&D Systems, USA) or isotype control IgG_2A_ (MAB006; R&D Systems, USA) 60 minutes prior to the HDM administration as indicated (Figure 1). The anti-TSLP mAb dose was predetermined by staining analysis of airway inflammation in mice that received 10–40 μg of anti-TSLP mAb, as previously described [24]. All of the mouse experimental procedures were performed in accordance with the guidelines of the Institutional Animal Care and Use Committee. The protocol was approved by the Committee on the Ethics of Animal Experiments of Vaccine Research Institute of Sun Yat-Sen University Vivarium.

### The measurement of airway hyperreactivity (AHR)

Twenty-four hours after the final HDM intranasal administration, The AHR was measured using whole-body plethysmography (Buxco Electronics, USA) as previously described, with slight modifications [25]. The mice were first placed in a chamber for acclimatization, the baseline response was determined, and then normal saline was nebulized to the mice, followed by the administration of increasing concentrations (6.25, 12.5, 25, 50 and 100 mg/ml, respectively) of nebulized methacholine (Sigma-Aldrich) in turn. The reading interval was set to 5 minutes following nebulization. The enhanced pause (Penh) values for each methacholine dose were determined using the following equation: [Penh =  pause× (peak inspiratory box flow/peak expiratory box flow)].

### Bronchoalveolar lavage fluid (BALF) and cell collection

The mice were sacrificed 24 hours after the assessment of the AHR. The left major bronchus was tied with a string, which was inserted with a 24-gauge needle, and the BALF was obtained by the infusion and collection of 0.5 ml of saline. The infusion and collection steps were repeated for 3 times. The BALF was centrifuged at 2,000 rpm for 10 minutes at 4°C, and the supernatant was stored at −80°C for further evaluation. The differential cell counts in the BALF were carried out as described [26]. In brief, the pellet was resuspended with 0.5 ml of saline and the differential cell counts were performed with Giemsa staining. The differential cell counts were determined by light microscopy from a count of at least 400 cells. The percentages of eosinophils, lymphocytes, and neutrophils were determined by counting their numbers in randomly selected high-power fields (40× magnifications) and by dividing this number by the total number of cells per high-power field. All of the counts were performed by the same observer in a blinded manner and in a randomized order at the end of the study.

### Flow cytometry

The BALF samples were centrifuged at 2,000 rpm for 10 minutes at 4°C, and the cell pellets were resuspended in a phosphate-buffered solution (PBS) to obtain single-cell suspensions. After counting, the single-cell suspensions were stained with combinations of PerCP-Cy 5.5-conjugated anti-mouse CD11c (45–0114), PE-conjugated anti-mouse OX40L (12–5905), FITC-conjugated anti-mouse CD80 (11–0801), and APC-conjugated anti-mouse CD86 (17-0862) antibodies, followed by staining with the appropriate fluorochrome-conjugated isotype control Ig. The antibodies were purchased from eBiosciences (eBiosciences, UK). The samples were incubated with the antibody cocktail for 20 minutes at room temperature, followed by centrifugation at 1,500 rpm for 5 minutes at 4°C. The cells were washed twice with PBS. After staining, the cells were resuspended in PBS and were analyzed by FACS LSRII (BD Biosciences, USA). The dead cells and debris were excluded based on light scatter properties. A total of 1×10^5^ to 3×10^5^ live events were acquired using FACS. The DCs were sorted as airway cells that expressed CD11c on FSC/SSC plots. The sorted CD11c+ populations were analyzed for the expression of the surface molecules OX40L, CD80 and CD86. Flow cytometry data acquisition was performed, and the data were analyzed, using BD FACSDiva software (BD Biosciences, USA).

### Elisa

The TSLP, TGF-β1, IL-4, IL-13 and IFN-γ concentrations in the BALF were measured with ELISA-kits (TSLP: R&D Systems, USA; TGF-β1, IL-4, IL-13 and IFN-γ: eBiosciences, UK). The protocols were followed according to the manufacturer's instructions. Briefly, the BALF samples were added in duplicate to 96-well plates with 100 μl per well. The appropriate biotin-conjugated antibodies were added to each well. The samples were incubated at room temperature for 2 hours. The wells were then aspirated, and each well was washed 5 times. The substrate solutions were added to each well, and were incubated for 30 minutes at room temperature in the dark. The optical density (O.D.) of each well was determined using a microplate reader (Bio-Rad Model 680, USA) that was set to 450 nm. A standard curve was created of the average of the O.D. duplicate readings. The results were calculated using Excel 2007(Microsoft office, USA).

### Histology

The lungs were dissected from the chest cavity after the lavage. The left lungs were immediately fixed in 4% paraformaldehyde and paraffin-embedded, and tissue sections (5 µm) were prepared. To assess airway remodeling, the sections were stained with periodic acid Schiff stain (PAS) for goblet cells or with Masson's trichrome for collagen, as described previously [27]. Briefly, goblet cell upregulation within the airway epithelia was assessed by measuring the length of the airway basement membrane that was covered by goblet cells. This measure was expressed as a percentage of the total basement membrane length in each airway. Three bronchioles were selected at random from each section; one section was analyzed per mouse and the mean goblet cell coverage (%) was calculated for each mouse. Peribronchial collagen thickness was measured using Image-Pro Plus software (version 6, Media Cybernetics, USA). Three bronchioles were selected at random from each section and the mean depth of collagen in the basement membrane was determined from five measurements around the bronchiole. One section was analyzed per mouse and the mean collagen depth was calculated for each mouse.

### Quantitative real-time PCR (RT-PCR)

The total RNA was extracted from the right lungs using Trizol (Invitrogen, USA). A reverse transcription-based quantitative PCR (real time PCR) was then performed to determine the mRNA levels from each RNA sample using a SYBR green-based DNA quantification method (Applied Biosystems, USA). The mRNA level of the β-actin gene was also determined for each RNA sample using RT-PCR. The reverse transcription assay was carried out using 2 μg of total RNA utilizing the protocol that was suggested by the manufacturer (Takara, Japan). The PCR procedure was performed using TaqMan Universal PCR master mix with 100 ng cDNA in a total volume of 20 μl. The PCR assays were completed using a CFX96 Detection System (Bio-Rad, USA) under the following conditions: 2 minutes at 94°C, followed by 40 cycles of 15 seconds at 95°C, 30 seconds at 56°C, and 60 seconds at 72°C. The sequences of each pair of primers were as follows: TSLP forward: 5′ -cggatggggctaacttaca-3′, reverse: 5′-tcctcgatttgctcgaactt-3′ [28]. T-bet forward: 5′-gccagccaaacagagaagac-3′, reverse: 5′-acacacctcctaccgaccag-3′ [29]; GATA-3 forward: 5′-gtggtcacactcggattcct-3′, reverse: 5′-gcaaaaaggagggtttaggg-3′ [30] and β-actin forward: 5′-tgctaggagccagagcagta-3′, reverse: 5′-agtgtgacgttgacatccgt-3′. TSLP, T-bet and GATA-3 mRNA expression levels for all of the treatment groups exposed to HDM were normalized to the levels that were determined from the lungs of mice exposed to saline. The RT-PCR data were analyzed using a comparative cycle threshold (C_t_) method, and the gene expression levels were calculated.

### Data analysis

The results from each group were compared using ANOVA with LSD tests, followed by post-testing with Dunn's multiple comparison of means. The coefficient of correlation was calculated using Spearman's correlation analysis. The statistical software package SPSS 16.0 for Windows was used for the analysis. The p values were less than 0.05 were considered to be statistically significant. All of the results are presented as the means±SEM.

## Results

### TSLP is upregulated in chronic HDM-exposed asthmatic mice airways

**Figure 2:**
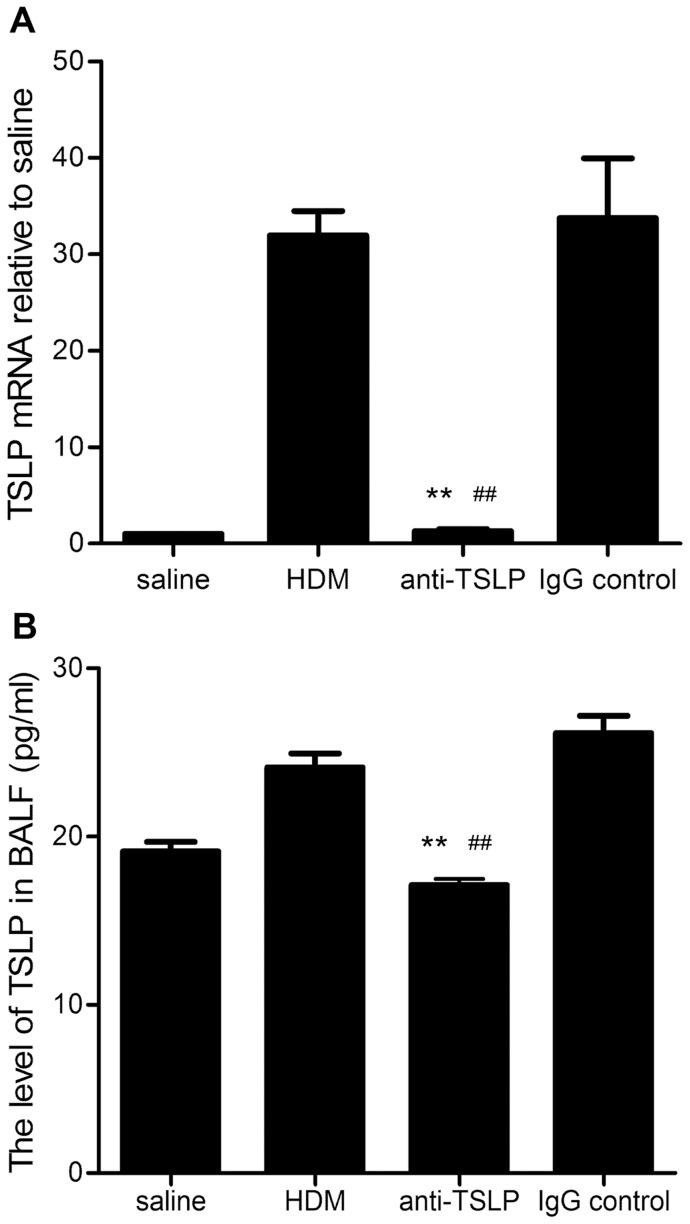
Chronic HDM exposure results in increased TSLP expression in mice. (A) The relative quantitative TSLP mRNA in the lung was detected using RT-PCR. (B) The TSLP protein concentration in the BALF was detected using an ELISA kit. The data shown represent the means±SEM (n = 5). mRNA value = 2^−ΔΔCt^, ΔΔCt =  (Ct_Target_-Ct_Actin_) of the treated mice-(Ct_Target_-Ct_Actin_) of the saline control mice. **p<0.01 compared with the HDM group. ^##^ p<0.01 compared with the IgG-treated control group. The results are derived from 4 experimental groups (5 mice/per group), and both the protein and mRNA levels were determined in duplicate.

We assessed TSLP expression in lung tissues to determine whether TSLP production increased in the airways of mice that were chronically exposed to HDM. As evaluated by ELISA and quantitative RT-PCR, both TSLP protein and mRNA expression levels were observed to be upregulated in the HDM-exposed mice (mRNA: 31.94±5.71 fold relative to saline-treated, and protein 24.12±1.82 pg/ml), compared with saline-treated control group (mRNA: 1, and protein 19.12±1.28 pg/ml). However, increased TSLP mRNA and protein levels in the lung were dramatically inhibited by pretreatment with anti-TSLP mAb (mRNA: 1.27±0.48 fold relative to saline-treated, and protein 17.12±0.84 pg/ml), compared with the levels that were observed in the IgG isotype-treated control mice (mRNA: 33.73±13.86 fold relative to saline-treated, and protein 26.16±0.84 pg/ml), even though the former group was continuously exposed to HDM from the 4^th^ week to the 5^th^ week (Figure 2A and 2B). These data indicated that prolonged HDM exposure resulted in TSLP overproduction in the airways of asthmatic mice.

### TSLP neutralization inhibits cellular inflammation and AHR to methacholine

**Figure 3:**
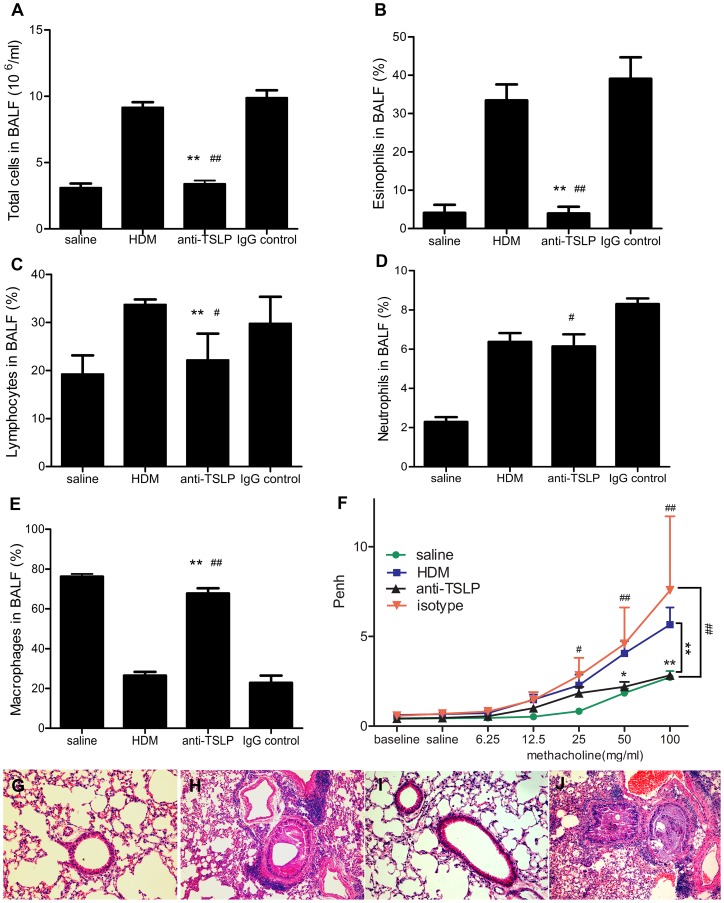
TSLP neutralization in the lung eliminates the airway inflammatory response and reduces methacholine-induced AHR. Number of total cells (A), eosinophils (B),lymphocytes(C),neutrophils (D) and macrophages (E) in the BALF revealed abundant inflammation in both the HDM-exposed mice and the IgG-treated controls, compared with the saline- and anti-TSLP-treated mice. The mice were intranasally administered HDM (5 days/week) for five consecutive weeks, and the airway inflammation was evaluated twenty-four hours following the AHR measurement. (F) Twenty-four hours after the final intranasal HDM or saline administration, the mice were stimulated with increasing doses of aerosolized methacholine (6.25, 12.5,25,50 and 100 mg/ml). AHR was measured using whole body plethysmography. (G-J) Representative photomicrographs of H&E-stained lung sections from mice that were exposed for 5 weeks to saline (G) or HDM (H) and pretreated with anti-TSLP mAb (I) or IgG isotype control (J) 60 minutes prior to and during the HDM exposure. Original magnification, 20×. The data shown represent the means±SEM (n = 5). *p<0.05 or **p<0.01 compared with the HDM group, ^#^ p<0.05 or ^##^ p<0.01 compared with the IgG-treated controls. The results are derived from 4 experimental groups (5 mice/per group) and the data are representative of 5 independent experiment.

Eosinophils are critical for the development of airway remodeling [31]. We showed that exposure to HDM for 5 consecutive weeks induced a robust airway inflammation, characterized by the influx of inflammatory cells, including eosinophils and lymphocytes (Figure 3H). Elevations in the total cell numbers, and the percentages of macrophages, eosinophils, lymphocytes and neutrophils (9.17±0.88×10^6^/ml, 26.47±4.20%, 33.43±4.16%, 33.73±1.06% and 6.37±1.0%, respectively) were observed in the BALF of HDM-exposed mice compared with the values that were observed in saline-treated controls (3.11±0.7×10^6^/ml, 76.21±3.02%, 4.11±2.06%, 19.28±3.87% and 2.29±0.54%, respectively) (Figure 3G). To determine whether blocking TSLP could reduce airway inflammatory cell infiltration, we pretreated mice with anti-TSLP mAb 60 minutes prior to weekly exposure to intranasal HDM from the 4^th^ week onward. We found that neutralization with anti-TSLP mAb significantly inhibited the elevation of the total cell numbers, as well as the percentages of macrophages, eosinophils, lymphocytes and neutrophils (3.4±0.55×10^6^/ml, 67.75±5.82%, 3.93±1.75%, 22.17±5.50% and 6.14±1.38%, respectively), compared with those of the IgG isotype-treated control mice (9.90±1.25×10^6^/ml, 22.84±8.05%, 39.06±5.61%, 29.80±5.53 and 8.29±0.65%, respectively) (Figure 3A–E, 3I and 3J). We additionally determined the effect of TSLP neutralization on AHR in the HDM model by assessing Penh. The mice were subjected to five weeks of continuous intranasal HDM exposure exhibited significantly increased responses and sensitivity to inhaled methacholine, compared with saline-treated control group. Treatment with the anti-TSLP mAb significantly inhibited the AHR to the similar levels of inhaled methacholine (Figure 3F). These data demonstrated that the local blockage of TSLP eliminated established airway eosinophilic inﬂammation and reduced the AHR to methacholine.

### TSLP neutralization inhibits airway structural remodeling

**Figure 4:**
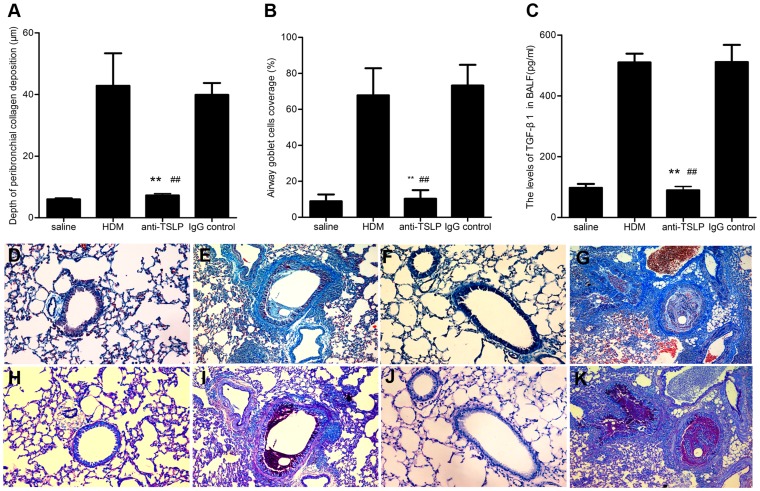
TSLP neutralization inhibits airway structural alterations. (A) The depth of peribronchial collagen deposition was significantly reduced in anti-TSLP-treated mice, compared with HDM-exposed and IgG isotype-treated controls. (B) Epithelial goblet cells were markedly reduced in anti-TSLP-treated mice, compared to the HDM-exposed and IgG -treated controls. (C) TGF-β1 levels in the BALF were significantly increased in HDM-exposed mice. The blockage of TSLP with the anti-TSLP mAb reduced TGF-β1levels in the BALF. TGF-β1 protein and mRNA levels were determined in duplicate experiments. (D-G) Representative photomicrographs of Masson's trichrome-stained lung sections from mice that were exposed for 5 weeks to saline (D) or HDM (E) and pretreated with anti-TSLP mAb (F) or an IgG-treated control (G) 60 minutes prior to and during HDM exposure. (H-K) Representative photomicrographs of airway sections of PAS-stained from mice that were exposed for 5 weeks to saline (H) or HDM (I) and pretreated with anti-TSLP mAb (J) or an IgG-treated control (K) 60 minutes prior to and during the HDM exposure. Original magnification, 20×. The data shown represent the means±SEM (n = 5), **p<0.01 compared with the HDM group, ^##^p<0.01 compared with the IgG-treated control mice. The photomicrographs of H-K are representative of 5 independent experiments.

The thickening of the airway wall is associated with peribronchial collagen deposition, and subepithelial fibrosis is a characteristic feature of airway remodeling [1]. We assessed airway peribronchial collagen deposition with Masson's trichrome stain. As shown in Figure 4E, repeated exposure to HDM for 5 consecutive weeks increased the thickness of airway walls, with more peribronchial collagen deposition being observed in the HDM-exposed mice (42.84±23.54 μm) than in the saline-treated controls (5.99±0.70 μm) (Figure 4A and 4D). Preventive neutralization of TSLP with the intranasal administration of anti-TSLP mAb during the period of HDM exposure dramatically attenuated the peribronchial collagen deposition (7.52±1.27 μm) (Figure 4A and 4F), reducing the thickness of the airway wall, compared with that in animals that were treated with the vehicle (39.87±8.62 μm) (Figure 4G). Goblet cell hyperplasia and metaplasia are common features of chronic asthmatic airways [9]. We next evaluated airway goblet cells and mucus with PAS staining. Continuous respiratory HDM exposure caused significant mucus hypersecretion in the luminar and goblet cell hyperplasia in the airway epithelia (67.83±33.69%) (Figure 4B and 4I), compared to saline-treated control mice (10.83±12.57%) (Figure 4H). The pretreatment with anti-TSLP mAb markedly inhibited mucus hypersecretion and goblet cell hyperplasia that was stimulated by prolonged HDM exposure (14.77±14.62%) (Figure 4J), compared with vehicle-treated mice (73.23±25.66%) (Figure 4K). These data demonstrated that TSLP overexpression in the lung was closely linked to chronic HDM exposure-induced airway remodeling. The local blockage of TSLP prevented airway structural remodeling by inhibiting peribronchial collagen deposition and goblet cell upregulation.

TGF-β1 plays an important role in the development of airway remodeling [32]. We therefore assessed whether anti-TSLP treatment could inhibit TGF-β1expression in the lung. The levels of TGF-β1 were significantly increased in mice that were subjected to chronic HDM exposure (509.77±64.65 pg/ml), compared with saline-treated controls (97.32±29.15pg/ml), whereas BALF TGF-β1 levels were significantly lower following pretreatment with anti-TSLP mAb (103.36±9.04 pg/ml), compared with IgG isotype-treated controls (511.09±126.35 pg/ml) (Figure 4C). The data revealed that downregulating TSLP production in the lung attenuated the level of the fibrosis-associated factor TGF-β1 in chronic HDM-exposed mice.

### TSLP neutralization modulates Th1/Th2 homeostasis

**Figure 5:**
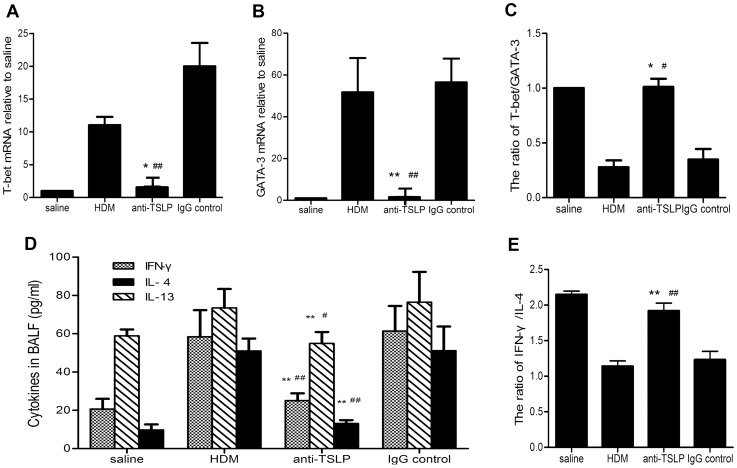
TSLP neutralization in the lung alters Th2-typed responses. The expression levels of T-bet mRNA (A) were elevated in all of the treatment groups following prolonged exposure to HDM for 5 weeks, whereas the expression levels of GATA-3 mRNA (B) were increased in the repeated HDM exposure group and the IgG-treated control mice. However, the T-bet/GATA-3 ratio (C) was lower in both the prolonged HDM exposure group and in the IgG-treated control mice, compared with both the anti-TSLP treatment group and the saline-treated control mice. The relative quantitative mRNA levels in the lung homogenate were detected using RT-PCR (D). The levels of the Th1-associated cytokine IFN-γ in the BALF were elevated in all of the subphenotype groups following exposure to HDM for 5 weeks. The levels of the Th2-associated cytokines IL-4 and IL-13 in the BALF were higher in the prolonged HDM-exposed group and in the IgG-treated control mice than in both the anti-TSLP mAb- and saline-treated control mice. However, IFN-γ/IL-4 ratio (E) was lower in both the prolonged HDM exposure group and the IgG-treated control mice than the ratio that was observed in the anti-TSLP mAb- and saline-treated control mice. The cytokines were elevated based on ELISAs. The data shown represent the means±SEM (n = 5), mRNA value = 2^−ΔΔCt^, ΔΔCt =  (Ct_Target_-Ct_Actin_) of the treated mice–(Ct_Target_-Ct_Actin_) of the saline control mice. * p<0.05 or ** p<0.01 compared with the HDM group. ^#^ p<0.05 or ^##^ p<0.01 compared with the IgG-treated controls. The results are derived from 4 experimental groups (5 mice/per group), and both protein and mRNA levels were determined in duplicate.

The homeostasis between T-bet and GATA-3 was previously associated with airway remodeling [10]. Th1 and Th2 cell differentiation is regulated by key transcriptional factors, such as T-bet for Th1 cell and GATA-3 for Th2 cell [11], [12]. We evaluated the expression levels of T-bet and GATA-3 mRNA in lung homogenate by RT-PCR to investigate the influence of TSLP on T-bet/GATA-3 homeostasis. The expression levels of T-bet mRNA were elevated in all of the subphenotypes that were subjected to prolonged (5 weeks) HDM exposure (11.03±2.79 fold in HDM group, 1.55±0.20 fold in anti-TSLP-treated group and 20.01±7.94 fold in IgG isotype-treated control group relative to saline-treated control group, respectively) (Figure 5A). Five weeks of HDM exposure contributed to a significant increase in GATA-3 mRNA expression in mice (51.73±36.67 fold relative to saline-treated controls). Pretreatment with anti-TSLP mAb significantly inhibited the evaluation of GATA-3 mRNA levels (1.55±0.20 fold relative to saline-treated controls), compared with IgG isotype-treated control mice (56.50±25.27 fold relative to saline-treated controls) (Figure 5B). Repeated exposure to HDM for 5 weeks contributed to a significant shift in T-bet/GATA-3 homeostasis, favouring GATA-3 expression (T-bet/GATA-3: 0.28±0.14), compared with saline-treated controls (the ratio of T-bet/GATA-3 served as 1). This bias was significantly reversed in mice that were intranasally administered anti-TSLP mAb (T-bet/GATA-3: 1.01±0.17 in anti-TSLP-treated group vs. 0.34±0.21 in IgG isotype -treated controls) (Figure 5C).

We analyzed the levels of Th1/Th2-associated cytokines in the BALF of the experimental animals to elucidate the contribution of anti-TSLP treatment to Th1/Th2 cytokine homeostasis in this mouse model. The IFN-γ level was elevated compared with that of saline-treated control animals (20.66±5.33 pg/ml) in all of the treatment groups following repeated HDM exposure (58.35±13.94 pg/ml in HDM group, 25.07±3.81 pg/ml in anti-TSLP-treated group and 61.42±13.50 pg/ml in IgG isotype-treated controls, respectively). The IL-4 level was increased in prolonged HDM-exposed mice (50.98±6.47 pg/ml), compared with saline-treated control animals (9.73±2.92 pg/ml). Anti-TSLP treatment significantly reduced IL-4 levels (13.14±1.72 pg/ml in anti-TSLP-treated mice compared to 51.11±12.64 pg/ml in IgG isotype-treated controls). The IL-13 level was also elevated in repeated HDM-exposed mice (73.45±9.88 pg/ml) compared with the saline-treated control animals (58.87±3.36 pg/ml). At the same time, the IL-13 level was lower in mice in which TSLP was blocked (54.89±5.98 pg/ml) than in the IgG isotype-treated control mice (76.43±15.85 pg/ml) (Figure 5D). For comparison, repeated exposure to HDM for 5 consecutive weeks led to a significant shift in the IFN-γ/IL-4 level ratio (IFN-γ/IL-4:1.14±0.17 in HDM-exposed mice vs. 2.15±0.11 in the saline-treated controls). This change, however, was significantly reversed by anti-TSLP mAb treatment ((IFN-γ/IL-4:1.92±0.25 in anti-TSLP-treated mice vs. 1.23±0.27 in the IgG isotype-treated control group) (Figure 5E). These findings clearly indicated that prolonged HDM exposure induced a robust Th2 response in mice, whereas downregulating the TSLP level in lungs with anti-TSLP mAb markedly suppressed the dominant production of Th2-associated cytokines and transcription factor and modulated Th1/Th2 homeostasis in an experimental chronic asthmatic model.

### TSLP neutralization inhibits the expression of the surface markers OX40L, CD80 and CD86 on airway CD11c+ cells

**Figure 6:**
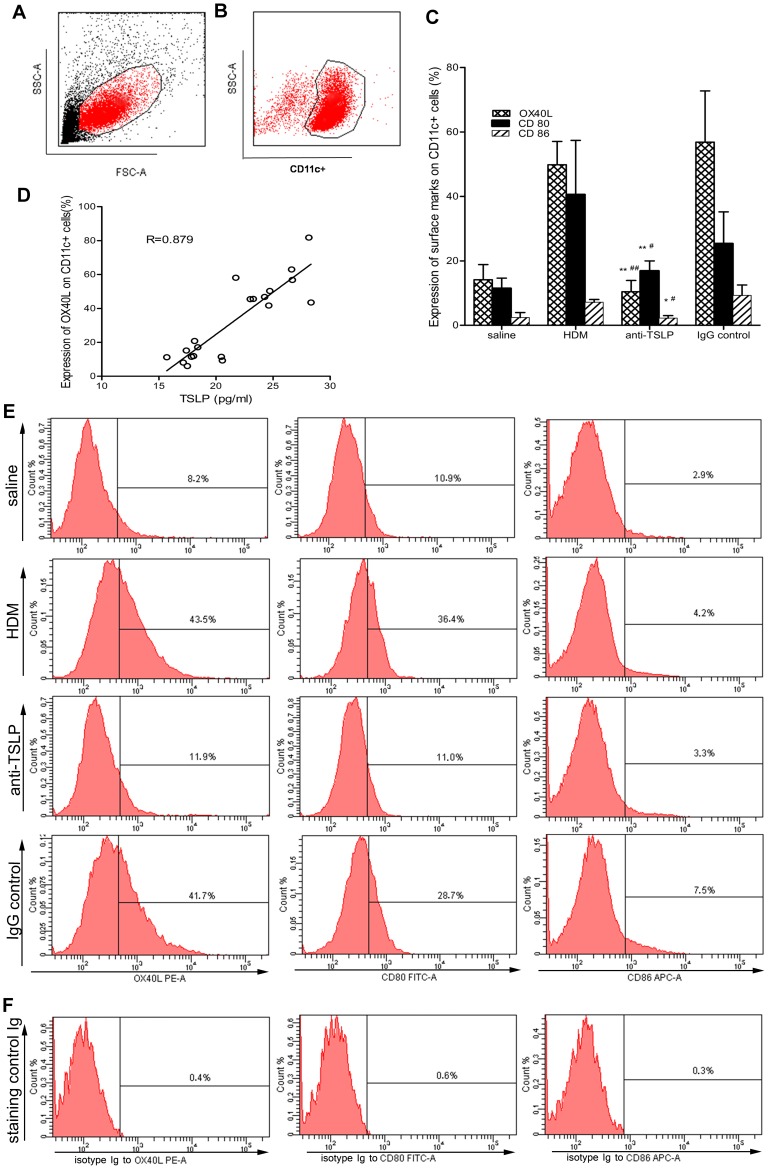
TSLP neutralization inhibits surface marker expression on airway CD11c+ cells. (A) Lung cell suspensions from mice were stained with combinations of PerCP-Cy 5.5-conjugated CD11c, PE-conjugated OX40L, FITC-conjugated CD80 and APC-conjugated CD86. The dead cells and the debris were excluded based on forward scatter (FSC)/side scatter (SSC) plots. (B) A total of 1×10^5^ to 3×10^5^ viable cells were acquired using FACS, and the DCs were then sorted as CD11c+ airway cells on FSC/SSC plots. (C) Mice that were chronically exposed to HDM received an intranasal administration of anti-TSLP mAb or of a control IgG 60 minutes prior to each HDM exposure. The expression levels of OX40L, CD80 and CD86 on pulmonary DCs were then analyzed using flow cytometry. The HDM-exposed mice exhibited a significant increase in the OX40L, CD80 and CD86 surface markers on CD11c+ airway cells. However, anti-TSLP pretreatment effectively reduced OX40L, CD80 and CD86 expression on the DCs, even though the mice were continuously exposed to HDM. (D) The TSLP levels in the BALF were highly correlated with OX40L expression on CD11c+ airway cells in all of the treatment mice (n = 20, R = 0.879, p<0.01). (E) Representative data from one of the five replicates. (F) Staining for OX40L (12-4031), CD80 (11-4888), and CD86 (17-4321) from isotype-Ig-treated controls. The data represent the means±SEM (n = 5). * p<0.05 or ** p<0.01 compared with the HDM group. ^#^ p<0.05 or ^##^ p<0.01 compared with the IgG-treated control mice. The results are derived from 4 experimental groups (5 mice/per group), and the data are representative of 5 independent experiment.

OX40L, which is expressed on activated-DCs, represented a key switch in the mediation of TSLP-induced Th2 responses by interacting with OX40 on T cells [17]. CD80 and CD86 were also identified salient surface markers that are highly expressed on mature DCs [33]. To clarify whether TSLP-primed naïve T cells differentiate into Th2 cells via the OX40L-OX40 pathway, we examined the expression of these molecules on CD11c+ airway cells using flow cytometry and evaluated the sensitivity of these expression levels to TSLP neutralization. We observed that the expression levels of OX40L, CD80 and CD86 on CD11c+ airway cells were upregulated after 5 weeks of HDM exposure (49.80±7.25%, 40.52±16.85%, and 7.16±2.84%, respectively), compared with the levels that were observed in saline-treated controls (14.12±4.70%, 11.44±3.20%, and 2.4±1.57%, respectively). The inhibition of TSLP with the anti-TSLP mAb for 2 weeks beginning on the 4^th^ week induced a marked downregulation in the expression levels of OX40L, CD80 and CD86 on CD11c+ airway cells (10.40±3.52%, 16.84±3.16%, and 2.22±0.77%, respectively), compared with the IgG isotype-treated controls (56.82±15.89%, 25.32±9.86%, and 9.3±3.21%, respectively) (Figure 6C). We next assessed the correlation between the level of TSLP and OX40L expression. As indicated in Figure 6D, the TSLP concentration in the BALF was highly correlated with OX40L expression on CD11c+ airway cells in all of the mice phenotypes. Given that CD11c is a marker of DCs, CD11c+ airway cells primarily represented airway DCs [34]. These results therefore suggested that repeated HDM exposure activated DCs and upregulated OX40L, CD80 and CD86 expression on mature DCs. The local administration of anti-TSLP mAb suppressed DC maturation, especially OX40L upregulation on DCs, and inhibited DC-mediated Th2 responses.

## Discussion

An emerging concept suggests that the airway epithelium is central to asthma pathogenesis [3], [13], [35]. As an epithelium-generated cytokine, TSLP is produced primarily by damaged epithelial cells and under specific conditions in the immune system to induce Th2 responses in allergic asthma [3], [36]. However, little is known about the influence of this cytokine on the development of the airway remodeling that is associated with chronic allergen-induced asthma. The aim of the present study was to investigate the effect of TSLP neutralization with an anti-TSLP mAb on the airway structural alterations in a murine model of chronic HDM exposure-induced asthma. In this mouse model, we demonstrated that the intranasal instillation of anti-TSLP mAb effectively inhibited airway eosinophil infiltration and AHR to methacholine, as well as produced a significantly preventive benefit with considerable improvement in airway structural remodeling.

Johnson et al. reported that the chronic exposure of mice to inhaled HDM for 5 days per week for up to 5 consecutive weeks stimulated a robust Th2 response, followed by high IL-4 and IL-13 levels, and airway remodeling. Such airway wall structural alterations were incipient after 3 weeks of exposure and striking after 5 weeks [23]. In this study, chronic asthmatic experimental mice were established using repeated, prolonged intranasal administration of HDM. To more accurately mimic the exposure to HDM experienced by asthmatic patients, no experimental adjuvant was used. (Figure 1). Consistent with the findings derived from previous experimental models [23], histological analysis of the chronic HDM-exposed asthmatic mice showed that repeated and continuous administration of HDM caused infiltration of eosinophils in the airway, thickened the basement membranes, upregulated goblet cells within the bronchial epithelia and increased the AHR to methacholine (Figure 3). Moreover, TSLP mRNA expression and protein production were significantly increased in this experimental asthmatic model (Figure 2), suggesting the possible involvement of TSLP in the development of airway remodeling.

Our data indicated that pretreatment with anti-TSLP mAb substantially alleviated established airway allergic inﬂammation such that there was only slight infiltration of total airway cells, eosinophils and lymphocytes and minimal mucus hyperplasia , as well as reduced AHR to levels similar to those resulting from methacholine inhalation (Figure 3). However, the number of lymphocytes in the BALF of all of the treatment groups was higher than that observed in naïve control group (data no shown). This result may be attributed to the effects of the various administrations on the lung which continuously stimulated by foreign substances, such as saline and inhaled anaesthetic agents. Infiltrating eosinophils in the airway were considered to contribute to the remodeling responses [31]. The profibrotic growth factor TGF-β1, which is expressed by eosinophils, played an important role in promoting the structural alterations associated with airway remodeling [37], [38]. In asthmatics, increased TGF-β1 mRNA expression levels were correlated with the depth of subepithelial fibrosis [39]. Anti-TGF-β1 mAb treatment was also demonstrated to alter the progression of airway remodeling following allergen challenge [40]. Furthermore, TGF-β1 is essential for the transformation of fibroblasts into myofibroblasts, which are major producers of collagen [41], [42]. Indeed, our data indicated that prolonged administration of HDM in mice caused eosinophil influx and peribronchial collagen deposition in the airway, an effect that is accompanied by increased TSLP and TGF-β1 levels. Strikingly, pretreatment with a high-affinity, potent, neutralizing anti-TSLP mAb led to a significant decrease in the number of airway eosinophils and TGF-β1 levels in mice during prolonged HDM exposure (Figure 4). These data implied that the blockage of TSLP reversed established airway inflammation and reduced both the production of TGF-β1 and peribronchial collagen deposition in chronic HDM-exposed mice.

Airway remodeling was primarily due to Th2 cell-mediated immune responses [7]–[10]. For example, mucous gland hyperplasia was observed to be dependent on Th2-typed inflammation in the airways of asthmatics. This effect was revealed by the findings that the adoptive transfer of Th2 cells promoted airway mucus expression and that the Th2 cytokines IL-4, IL-9 and IL-13 induced goblet cell hyperplasia in the lung [43]. Moreover, we previously demonstrated that IL-13 levels are negatively correlated with the outcome of lung function in children with allergic asthma [44]. A recent study showed that neutralization of IL-13 reduced airway inflammation, improved baseline lung function and inhibited airway remodeling in a mouse model of allergic asthma [27]. Consistent with these previous observation, we demonstrated that prolonged HDM exposure induced a robust Th2 response, followed by high levels of IL-4 and IL-13. Furthermore, the local blockage of TSLP significantly reduced the levels of Th2-associated cytokines (Figure 5). Importantly, the mice that were pretreated with anti-TSLP mAb, failed to develop the salient features of airway remodeling, such as peribronchial collagen deposition, goblet cell hyperplasia and mucus overproduction, following prolonged exposure to HDM extracts (Figure 4).

T-bet and GATA-3 are believed to be the master regulators of Th1 and Th2 cell differentiation and to specifically function in helper T cells [11], [12]. A study that used transgenic mice that overexpress GATA-3 revealed that the pathogenesis of airway remodeling was caused by Th2 responses, which depended on a shift of T-bet/GATA-3 homeostasis in the lung toward GATA-3 activity [10]. Shi L et al. demonstrated that, compared with mice that received isotype Ig controls, the local administration of anti-TSLPR antibody in OVA-induced asthmatic mice resulted in a significant reduction in the levels of the Th2-associated cytokines IL-4 and IL-5 while enhancing the expression of the Th1-associated cytokine IFN-γ. This previous study implied that TSLP signaling played a pivotal role in Th1/Th2 homeostasis [22]. Murai H et al. further revealed that aeroantigens like alternaria rapidly induced damage to the epithelium, releasing cytokines that promoted Th2 differentiation of naïve T-cells by increasing GATA3 expression [3]. To further address the effects of TSLP blockage on HDM-induced Th2 responses, we compared the expression of T-bet and GATA-3 in lung tissues that were isolated from the different experimental groups. As expected, GATA-3 was overexpressed in HDM-induced asthmatic mice, and the T-bet/GATA-3 homeostasis was shifted to GATA-3. More strikingly, anti-TSLP administration appeared to remodel T-bet/GATA-3 homeostasis by significantly decreasing GATA-3 expression in addition to moderately downregulating T-bet expression. This result was consistent with the cytokine profile that we observed in the BALF of anti-TSLP-treated asthmatic mice and highlighted the important role of TSLP-mediated signaling in the conditioning of the homeostasis of the Th1/Th2 responses.

Our data differ in part from the findings of other groups. Shi L et al. demonstrated that treatment with anti-TSLPR significantly reduced Th2 responses by decreasing the expression of IL-4 and IL-5 in addition to moderately elevating IFN-γ in a mouse model of OVA-induced asthma [22]. However, our data demonstrated that prolonged HDM exposure induced a robust Th2 response that was accompanied by a marked elevation of IFN-γ levels. Pretreatment with anti-TSLP mAb dramatically suppressed Th2 responses by decreasing the levels of Th2-associated cytokines, such as IL-4 and IL-13, and the Th2-associated transcription factor GATA-3. A moderate downregulation of the expressions of the Th1-associated cytokine IFN-γ and the Th1-associated transcription factor T-bet was also observed. The observed difference in IFN-γ expression levels between our study and previous studies was most likely attributable to the distinct endpoint and the animal model used in the different studies. A recent study indicated that IFN-γ plays a key role in the progression of tissues fibrosis and remodeling [45]. The neutralization of IFN-γ during antigen challenge with antigen plus IL-18 inhibited both lung fibrosis and periostin deposition and both neutrophilic infiltration and airway hyperresponsiveness, respectively [46]. Shi L et al. focused on the effects of the local blockade of the TSLP receptor on acute OVA-induced airway inflammation [22], whereas our research aimed to examine the role of TSLP in HDM-induced airway remodeling, which is characterized by subepithelial fibrosis and peribronchial collagen deposition, ultimately resulting in the upregulation of IFN-γ expression in the lung. However, it should be emphasized that, although IFN-γ is highly expressed in HDM-exposed mice, the homeostasis of Th1/Th2 cytokines remained shifted to Th2 compared with the saline-treated controls (Figure 5).

T cell activation requires the antigen that is presented by the antigen-presenting cell (APC) to be recognized by the T cell receptor [47], [48]. Acting as a potent APC, DCs play a crucial role in bridging innate and adaptive immunity in the lung [49]. TSLP represents a key mediator that links epithelial cells and DCs at the interface of allergic inflammation by participating in the programming of DC-mediated Th2 polarization [50], [51]. TSLP was revealed to initiate the development of Th2 immune responses through its activation of CD11c+ DCs and to upregulate OX40L. OX40L then interacts with OX40 on T cells, priming naïve T cell differentiation into Th2 effector cells and causing the secretion of Th2-attracting chemokines [22], [50]–[52]. In our model, given that chronic HDM-exposure contributed to the overproduction of TSLP, we addressed whether TSLP affected the activation of airway DCs. We determined DC surface marker expressions using flow cytometry. Not unexpectedly, we found that chronic HDM exposure induced immature DC (iDC) maturation, with significantly higher expression of DC surface molecules CD80 and CD86 compared with the expression of these molecules in saline-treated controls (Figure 6). Interestingly, pretreatment with anti-TSLP mAb dramatically inhibited OX40L, CD80 and CD86 expression on DCs, even though the mice were continuously exposed to HDM. We previously demonstrated that TSLP was overproduced in the BALF and that TSLPR was highly expressed on lung DCs in asthmatic mice compared with saline-treated controls [53]. This knowledge was extended by the present study, which revealed that high TSLP expression in the lung was associated with increased OX40L expression on CD11c+ airway cells (Figure 6D). Together, these data could serve as an explanation for the epithelium-derived TSLP activation of airway DCs, which in turn upregulate OX40L, via OX40L-OX40 pathways, priming naïve T cells to differentiate into Th2 cells. However, we can derive only limited conclusions from our results. Further studies are required to confirm these findings and to precisely define the relevant pathways and the other effects of TSLP on airway structural cells using ex vivo experiments and in vivo chronic HDM-exposed models.

In conclusion, our data demonstrated that continuous exposure to aeroantigens damaged epithelial cells and resulted in TSLP overexpression in the airway. TSLP conditioned the immune system to induce a robust Th2 response, initiating chronic airway inflammation and structural remodeling in mice with chronic asthma. The local neutralization of TSLP significantly reversed airway eosinophilic inflammation; reduced peribronchial collagen deposition, goblet cell hyperplasia and mucus overproduction; and decreased both AHR and TGF-β1 levels in chronic HDM-exposed mice. These findings provide evidence that the epithelium-generated cytokine TSLP, at least in part, is an important factor in the initiation and persistence of both airway inﬂammation and structural remodeling. Our study suggested that downregulating TSLP levels in asthmatic airway may benefit patients with chronic allergic asthma by reducing airway inflammation and improving lung function, with the potential to inhibit airway remodeling. Thus, protecting epithelial cells from damage may be a novel therapeutic strategy to treat allergic asthma.
